# Antimicrobial resistance and genomic characterization of *Salmonella enterica* serovar Senftenberg isolates in production animals from the United States

**DOI:** 10.3389/fmicb.2022.979790

**Published:** 2022-11-03

**Authors:** Mariela E. Srednik, Brenda R. Morningstar-Shaw, Jessica A. Hicks, Tonya A. Mackie, Linda K. Schlater

**Affiliations:** National Veterinary Services Laboratories, Animal and Plant Health Inspection Service, United States Department of Agriculture, Ames, IA, United States

**Keywords:** antimicrobial resistance, whole genome sequencing, phylogeny, resistance genes, *Salmonella* Senftenberg

## Abstract

In the USA, *Salmonella enterica* subspecies *enterica* serovar Senftenberg is among the top five serovars isolated from food and the top 11 serovars isolated from clinically ill animals. Human infections are associated with exposure to farm environments or contaminated food. The objective of this study was to characterize *S.* Senftenberg isolates from production animals by analyzing phenotypic antimicrobial resistance profiles, genomic features and phylogeny.

*Salmonella* Senftenberg isolates (n = 94) from 20 US states were selected from NVSL submissions (2014–2017), tested against 14 antimicrobial drugs, and resistance phenotypes determined. Resistance genotypes were determined using whole genome sequencing analysis with AMRFinder and the NCBI and ResFinder databases with ABRicate. Plasmids were detected using PlasmidFinder. Integrons were detected using IntFinder and manual alignment with reference genes. Multilocus-sequence-typing (MLST) was determined using ABRicate with PubMLST database, and phylogeny was determined using vSNP.

Among 94 isolates, 60.6% were resistant to at least one antimicrobial and 39.4% showed multidrug resistance. The most prevalent resistance findings were for streptomycin (44.7%), tetracycline (42.6%), ampicillin (36.2%) and sulfisoxazole (32.9%). The most commonly found antimicrobial resistance genes were *aac*(6′)-Iaa (100%), *aph*(3″)-Ib and *aph*(6)-Id (29.8%) for aminoglycosides, followed by *bla*_TEM-1_ (26.6%) for penicillins, *sul*1 (25.5%) and *sul*2 (23.4%) for sulfonamides and *tet*A (23.4%) for tetracyclines. Quinolone-resistant isolates presented mutations in *gyr*A and/or *par*C genes. Class 1 integrons were found in 37 isolates. Thirty-six plasmid types were identified among 77.7% of the isolates. Phylogenetic analysis identified two distinct lineages of *S.* Senftenberg that correlated with the MLST results. Isolates were classified into two distinct sequence types (ST): ST14 (97.9%) and ST 185 (2.1%). The diversity of this serotype suggests multiple introductions into animal populations from outside sources.

This study provided antimicrobial susceptibility and genomic characteristics of *S.* Senftenberg clinical isolates from production animals in the USA during 2014 to 2017. This study will serve as a base for future studies focused on the phenotypic and molecular antimicrobial characterization of *S.* Senftenberg isolates in animals. Monitoring of antimicrobial resistance to detect emergence of multidrug-resistant strains is critical.

## Introduction

*Salmonella enterica* subsp. *enterica* serovar Senftenberg is commonly isolated from animals and food. This serovar is widely distributed and has been found worldwide. In the USA, *S*. Senftenberg is among the top five serovars isolated from food and among the top 11 serovars isolated from clinically ill animals (Switt, 2019). *S.* Senftenberg and *S*. Montevideo were the most common serotypes found in animal feeds in the USA in 2012 (Li et al., 2012). In Europe, *S.* Senftenberg was found more frequently in poultry flocks than other serotypes and the emergence of this serotype was a cause of concern in 2012 (Boumart et al., 2012). Most *Salmonella* serovars, including *S.* Senftenberg, are tolerant to desiccation and able to colonize and persist in feed mills (Pedersen et al., 2008). Interestingly, *S.* Senftenberg is also a heat-resistant serotype (Doyle and Mazzotta, 1999), which may contribute to persistence in feed and the environment. This can be a source of contamination on farms and in processing environments. Human infections with *S*. Senftenberg are rare and are typically associated with exposure to poultry flocks, farm environments, or contaminated food (Boumart et al., 2012). Worldwide, *S*. Senftenberg has been linked to outbreaks associated with contaminated pistachios, salami, basil, Maradol papayas, peanut butter, alfalfa sprouts and baby cereal (Centers for disease Control and Prevention, 2010, 2016, 2022; US Food and Drug Administration, 2014; Hassan et al., 2019; Switt, 2019; Haendiges et al., 2021).

Although antimicrobial-resistant isolates of *S.* Senftenberg are usually associated with animal sources (Stepan et al., 2011), antimicrobial-resistant human isolates of *S*. Senftenberg have been reported in the USA, and extensively drug-resistant (XDR) strains have been isolated from patients outside of the USA, raising public health concerns (Hendriksen et al., 2013; Veeraraghavan et al., 2019). Infections caused by *S*. Senftenberg range from asymptomatic to severe, and deaths have been associated with the XDR strains of *S*. Senftenberg in China (El Ghany et al., 2016). XDR strains are resistant to all but only one or two categories of antimicrobials, leaving clinicians and veterinarians with few to no treatment options (Magiorakos et al., 2012).

Horizontal transfer of genetic material is important in the spread of MDR. Resistance genes can be inserted in the form of cassettes into integrons; these mobilizable genetic elements are grouped in three classes (class 1, 2 and 3) based on the presence of three different integrases encoded by the *intl*1, *intl*2, *intl*3 genes. Integrons can be mobilized within the chromosome to other regions, or they can be inserted in integrative-conjugative elements and plasmids, which can facilitate the horizontal transfer of resistance genes between bacteria (Stokes and Gillings, 2011).

Since *Salmonella* is associated with outbreaks of foodborne disease, MDR strains pose a risk to public health because of the potential for treatment failures (Nair et al., 2018). Few studies exist on the antimicrobial susceptibility and genetic diversity of *Salmonella* serovar Senftenberg of animal origin. Among all *Salmonella* serotyping submissions received at the NVSL from January 1, 2014, through December 31, 2017, *S.* Senftenberg ranked number eight during 2014 and number 10 during 2015 among clinical isolates, and ranked number one during 2014, 2015, 2016 and number two during 2017 among non-clinical isolates. The objective of this study was to compare phenotypic and genomic resistance data, mechanisms of antimicrobial resistance, plasmid replicons, genetic relatedness, and to characterize *S.* Senftenberg diagnostic isolates recovered from poultry, swine, and cattle in the USA between 2014 and 2017, and to provide useful retrospective information for future studies on *S.* Senftenberg.

## Materials and methods

### Bacterial isolates

A total of 94 *S.* Senftenberg isolates from swine (n = 50), poultry (n = 24) and cattle (n = 20) were selected from the National Veterinary Services Laboratories (NVSL) *Salmonella* repository isolates archived at room temperature on nutrient agar slants. Samples came from 20 US States (IA = 20, MN = 15, AR = 8, MO = 8, IL = 6, IN = 4, TX = 4, NC = 4, OH = 4, PA = 4, OK = 2, KS = 2, NE = 2, NY = 2, SD = 2, VA = 2, WI = 2, AL = 1, AZ = 1, KY = 1).

Isolates were selected from samples that were submitted to the NVSL for *Salmonella* serotyping, confirmed by classical (Grimont and Weill, 2007) and molecular typing using Luminex xMAP® technology (Dunbar et al., 2015), between the years of 2014 and 2017. The dataset was initially limited to one sample per year per owner. If more than the targeted number of isolates were available, a randomly selected subset of isolates was chosen. The data was then de-identified to remove information other than the animal species, state of origin, clinical status, and sample type and assigned a unique identifier. *Salmonella* was confirmed using Biotyper software with an autoflex speed™ MALDI-TOF instrument (Bruker Daltonics, Billerica, MA, USA).

### Antimicrobial susceptibility testing

All *Salmonella* isolates were tested for antimicrobial susceptibility against 14 class-representative antimicrobial agents using the Sensititre CMV4AGNF plate (Thermo Fisher Scientific, Waltham, MA, USA) including: gentamicin (GEN), streptomycin (STR), amoxicillin/clavulanic acid (AMC), cefoxitin (FOX), ceftriaxone (CRO), meropenem (MEM), sulfisoxazole (SUL), trimethoprim/sulfamethoxazole (SXT), ampicillin (AMP), chloramphenicol (CHL), ciprofloxacin (CIP), nalidixic acid (NAL), azithromycin (AZM), and tetracycline (TET). Results were interpreted using consensus interpretative criteria established by the National Antimicrobial Resistance Monitoring System (US Food and Drug Administration, 2021).

### Whole genome sequencing and genome analysis

DNA was extracted using Promega Maxwell® with the Whole Blood DNA kit following manufacturer’s instructions. *S.* Senftenberg isolates were subjected to whole genome sequencing using the Illumina MiSeq platform with 2×250 paired-end chemistry and the NexteraXT library preparation kit (Illumina, Inc., San Diego, CA, USA). AMR gene alleles were determined using AMRFinder (Feldgarden et al., 2019) and the NCBI and ResFinder databases (Zankari et al., 2012) using ABRicate^1^ with an identity threshold of 80% over ≥60% of the length of the target gene. Integrons were identified using IntFinder 1.0 (Loaiza et al., 2020). Integron classes were mapped using NCBI reference sequences of the *intl*1 (MG785026.1), *intl*2 (MK994977.1) and *Intl*3 (KM194584.1) integrase genes, and resistance genes available in the CARD (Comprehensive Antimicrobial Resistance Database, card.macmaster.ca) database using Geneious Prime v11.0.9 + 11 (Biomatters Ltd., NZ). Plasmid replicons were identified using ABRicate with the PlasmidFinder database (Carattoli et al, 2014). PointFinder was used for analysis of chromosomal structural gene mutations (Zankari et al., 2017). Isolate sequences are publicly available in the NCBI BioProject PRJNA785813.

Multilocus-sequence-typing (MLST) was determined using ABRicate with PubMLST database. The single nucleotide polymorphism (SNP) analysis of all isolates was performed using the NVSL vSNP pipeline^1^. Isolates were separated and analyzed by MLST with the respective reference (*S.* Senftenberg NZ_CP016837 for ST185 and NZ_CP029036 for ST14). A SNP-based phylogenetic tree was generated with RAxML in the vSNP pipeline (Stamatakis, 2014). The k-mer based phylogeny tool kSNP was used to generate a reference-free phylogenetic tree of all the *S*. Senftenberg isolates (Gardner et al., 2015).

### Relationship of antimicrobial susceptibility with antimicrobial genes

Each antimicrobial susceptibility interpretation (resistant or susceptible) for each antimicrobial tested was compared with the presence or absence of the corresponding resistance gene or genes and/or chromosomal gene mutations found. Intermediate phenotypes were counted as susceptible in this analysis. Using the phenotypic results as the reference outcome, sensitivity was calculated by dividing the number of isolates that were genotypically resistant by the total number of isolates exhibiting clinical resistance phenotypes. Specificity was calculated by dividing the number of isolates that were genotypically susceptible by the total number of isolates with susceptible phenotypes (McDermott et al., 2016).

## Results

### Antimicrobial susceptibility testing

**Figure 1:**
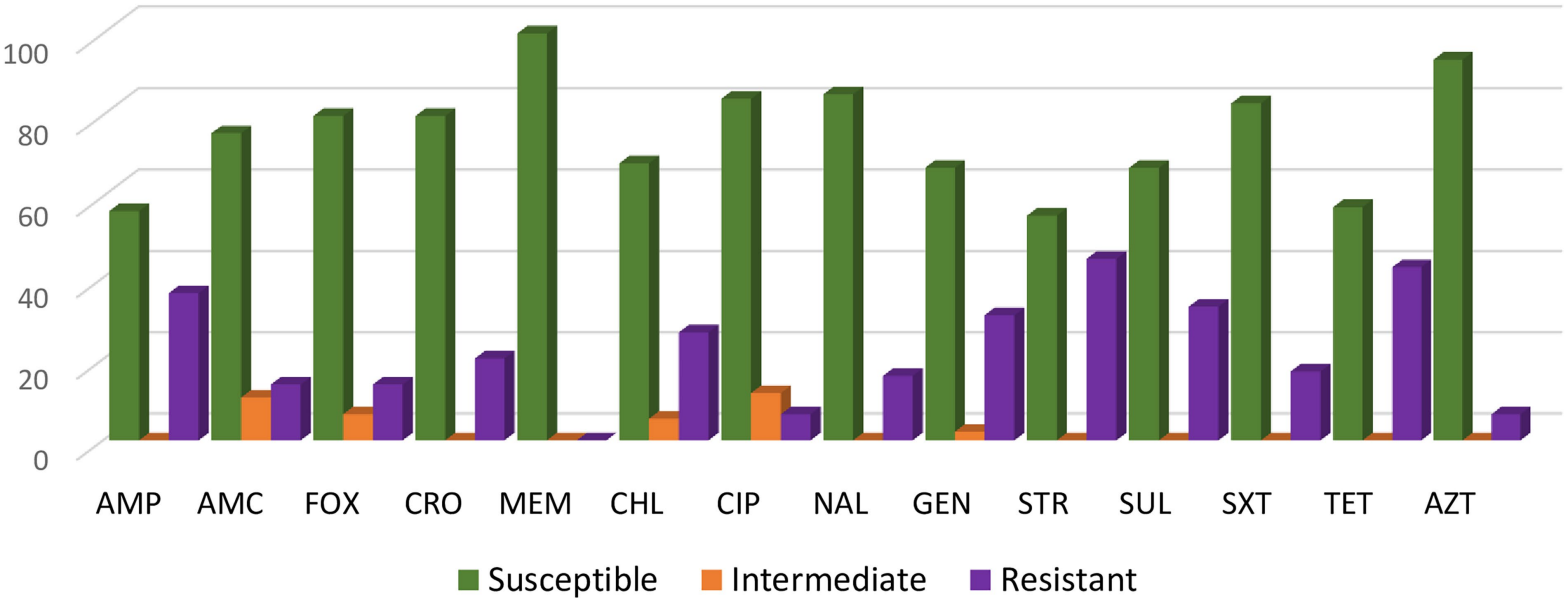
Antimicrobial resistance rates of the 94 *Salmonella* Senftenberg isolates. For ciprofloxacin (CIP) isolates categorized as intermediate (MIC 0.12 to 0.5 μg/mL) or resistant (MIC ≥ 1 μg/mL) were defined as having decreased susceptibility to CIP with a MIC ≥ 0.12 μg/mL.

Overall, the highest percentage of resistance was found to the following antimicrobials: streptomycin (44.7%), tetracycline (42.6%), ampicillin (36.2%) and sulfisoxazole (32.9%) (Figure 1). All of the *S.* Senftenberg isolates were susceptible to meropenem.

Among all 94 isolates, 60.6% (n = 57) were resistant to at least one antimicrobial and 39.4% (n = 37) isolates were MDR. Among MDR isolates, 60% were from swine, 20% from cattle and 12.5% from poultry. Four isolates from swine showed possible XDR; one isolate was only susceptible to meropenem, one isolate was susceptible to gentamycin and meropenem, and two isolates were susceptible to meropenem and azithromycin (one of which with presence of the macrolide *erm*B gene). Most isolates showed diverse resistance profiles, with the most common resistance profile (AMP, GEN, STR) found in just five isolates. Supplementary Table S1 summarizes the phenotypic resistance profiles and the resistance genes present in *S.* Senftenberg isolates displaying antimicrobial resistance.

### Antimicrobial resistance genes and integrons

Antimicrobial resistance genes are shown in Table 1. The most commonly observed was the aminoglycoside acetyltransferase *aac*(6′)-Iaa gene, which was found in all isolates, and the aminoglycoside phosphotransferase *aph*(3″)-Ib and *aph*(6)-Id genes seen together in 29.8% (n = 28) of isolates. The *sul*1 gene, a dihydropteroate synthase that is linked to other resistance genes of class 1 integrons and confers resistance to sulfonamides, was found in 25.5% (n = 24) of the isolates; and the *sul*2 gene was observed in 23.4% (n = 22) of the isolates. Resistance to tetracycline was due to the presence of the *tet*A gene that encodes a tetracycline efflux pump, and it was observed in 23.4% (n = 22) of the isolates.

**Table 1:** Antimicrobial resistance genes of the 94 *Salmonella* Senftenberg isolates.

Drug classes	Resistance genes
β-lactams	*bla*_TEM-1B_ (26.6%), *bla*_CMY-2_ (14.9%), *bla*_SHV-12_ (5.3%), *bla*_TEM-1A_ (1.1%)
Phenicols	*flo*R (14.9%), *cat*A2 (8.5%), *cml*A5 (3.4%), *cml*A1 (1.1%).
Quinolones	*qnr*B2 (6.4%), *qnr*B19 (3.4%), *qnr*B77 (2.1%), *qnr*B6 (1.1%).
Quinolones/Aminoglycosides	*aac*(6′)-Ib-cr (1.1%).
Aminoglycosides	*aac*(6′)-laa (100%), *aph*(3″)-Ib (29.8%), *aph*(6)-Id (29.8%), *aph*(3′)-Ia (23.4%), *aad*A1 (17%), *aad*A2 (15.9%), *aac*(6′)-Ib4 (9.6%), *aac*(6′)-Ib (7.4%), *aac*(6′)-IIc (7.4%), *aac*(3)-II (7.4%), *aac*(3)-*VIa* (7.4%), *ant*(2″)-Ia (7.4%), *aad*A6 (3.4%), *aph*(4)-Ia (3.4%), and others with 1.1%.
Folate pathway inhibitors	*sul*1 (25.5%), *sul*2 (23.4%), *dfr*A19 (6.4%), *dfr*A1 (3.4%), *dfr*A34 (3.4%), *dfr*A12 (2.1%), *dfr*A15 (2.1%), *dfr*A27 (1.1%), *sul*3 (1.1%)
Tetracyclines	*tet*A (23.4%), *tet*B (8.5%), *tet*D (8.5%), 5.3%), *tet*X (1.1%).
Macrolides	*ere*A (6.4%), *mph*A (3.2%), *erm*42 (2.1%), *mph*E (1.1%), *erm*B (1.1%), *msr*E (1.1%).
Ansamycins (Rifamycin)	*arr-*269,927,220 (7.4%), *arr*-3 (1.1%).
Glycopeptides (Bleomycin)	*ble*O (3.2%), *ble*Tn5 (1.1%).
Polymyxins (Colistin)	*mcr*-9.1 (8.5%)

Among beta-lactams, *bla*_TEM-1B_, a class A narrow-spectrum beta-lactamase, was the most prevalent beta-lactamase gene (26.6%) conferring resistance to penicillins (ampicillin); but *bla*_CMY-2_, a class C beta-lactamase_,_ was the most prevalent beta-lactamase gene (14.9%) against penicillins plus inhibitors (amoxicillin + clavulanic acid) and cephalosporins (cefoxitin, ceftriaxone). The class A extended spectrum beta-lactamases (ESBLs) encoded by the *bla*_SHV-12_ gene were found in five isolates from swine.

In addition to *aph*(3″)-Ib and *aph*(6)-Id genes, other resistance genes were observed that convey resistance to aminoglycosides, including several integron-encoded aminoglycoside nucleotidyltransferases such as *aad*A1, which was observed in 17% of isolates, *aad*A2, which was observed in 15.9% of isolates, and *aad*A5, *aad*A6, *aad*A12, *aad*A16, and *aad*A25, which were observed in lower frequencies. Resistance genes for gentamicin included several aminoglycoside acetyltransferases encoded by: *aac*(6′)-Ib4 gene in 9.6%; *aac*(6′)-Ib, *aac*(3′)-II, *aac*(6′)-IIc, *aac*(3)-VIa in 7.4%; and *aac*(3)-IVa in 4.3%. The nucleotidyltransferase *ant*(2″)-la gene was found in 7.4%, and the methyltransferase *arm*A gene in one isolate. Six genes conferring resistance to macrolides were found in 13.8% of isolates; the *ere*A gene that encodes an erythromycin esterase was the most frequently identified in 6 isolates, followed by the *mph*A gene that encodes a macrolide 2′-phosphotransferase in 3 isolates. Several resistance genes were found for chloramphenicol; the most frequent was the plasmid or transposon-encoded chloramphenicol exporter *flo*R, which was observed in 14.9% of isolates, followed by *cat*A2 gene, a chloramphenicol O-acetyltransferase, which was observed in 8.5% of isolates. Additional chloramphenicol exporter genes found in lower frequency were *cml*A5 (3.4%) and *cml*A1 (1.1%) genes.

Quinolone-resistance genes were detected in 12 isolates; the most frequent gene was *qnr*B2, a plasmid-mediated quinolone resistance protein, which was observed in 6.4% of isolates, and others in lower frequency: *qnr*B19 in 3.4%, *qnr*B77 in 2.1%, *qnr*B6 in 1.1% and *aac*(6′)-lb-cr in 1.1% of isolates. The *aac*(6′)-lb-cr gene doubly confers resistance to aminoglycoside and fluoroquinolone antibiotics through fluoroquinolone-acetylating activity.

Some isolates presented other genes that confer resistance to antimicrobials that were not included on the panel: the *aph*(3′)-Ia gene conferring resistance to kanamycin was detected in 22 (23.4%) isolates, the mobilized and plasmid-mediated colistin resistance and phosphoethanolamine transferase *mcr*-9.1 gene was detected in eight (8.5%) isolates, the *arr-*269927220, an ADP-ribosyltransferase that confers resistance to rifamycin was detected in seven (7.4%) isolates, and the *arr*-3 gene in one (1.1%) isolate. The *ble*O gene that encodes a bleomycin binding protein was detected in three (3.2%) isolates, and the *ble*Tn5 gene that encodes a bleomycin binding protein BLMT by the *ble* gene on the transposon Tn5 was found in one (1.1%) isolate. Interestingly, the *aac*(6′)-Iaa gene was observed in all isolates using ResFinder databases. The *aac*(6′)-Iaa gene is a chromosomal-encoded aminoglycoside acetyltransferase that confers resistance to tobramycin and kanamycin aminoglycosides. The gene resistance profile varied among the isolates. In addition to antimicrobial resistance genes, we observed the gene *qac*EΔ1, which confers resistance to quaternary ammonium compounds (QAC), in 25 (26.6%) isolates. We also detected the presence of a sulfonamide resistance gene and the presence of the *intl*1 gene, a class 1 integron, in these isolates (Table 2). Among isolates that showed resistance to at least one antimicrobial, 64.9% (n = 37) were positive for *intI*1, but no isolates carried *intI*2 or *intI*3 genes. Class 1 integrons variable regions enclosed one or several gene cassettes containing *aad*A, *drf*A, *flo*R, *aac*(6′)-Ib, *ant*(2′′)-la, and *cml*A. Class 1 integrons were found in different proportions among species source: 78.6% (n = 11) in poultry, 61.1% (n = 22) in swine and 47.1% (n = 4) in cattle isolates (Table 3). The *sul*1 gene, which is often carried in the conserved sequence (3′ CS) of a class 1 integron, was missing in 11 isolates. Ten of these integrons were identified as In48, and carried a resistant gene cassette (*aac*(6)-Ib), the other one was classified as In192, positive for *Intl*1 and carried a *dfr*A15 cassette.

**Table 2:** Antimicrobial resistance genes detected in the variable region of class 1 integrons among *Salmonella* Senftenberg isolates from the USA.

Source					Isolate ID	Integrase	Gene cassettes in the variable region	3’ CS	IntFinder 1.0				Integron name	Identity (%)	Query/template length (bp)	Accession number
Swine	18–006979-062	*Intl*1	*aad*A2	*qac*EΔ1, *sul*1	In128	99.9	1,009/1,009	AF221903
Swine	18–006979-143	*Intl*1	-	*qacE*Δ1, *sul*1	-			
Swine	18–006979-166	*Intl*1	*ant*(2″)-Ia, *cml*A5	*qac*EΔ1, *sul*1	In571	98.3	3,016/2,999	AB285479
Swine	18–006979-167	*Intl*1	*aad*A1	*qacE*Δ1, *sul*1	-			
Swine	18–006979-173	*Intl*1	-	*qacE*Δ1, *sul*1	-			
Swine	18–012180-030	*Intl*1	*dfr*A15, *aad*A1, *aac*(3)-*VIa*	-	-			
Swine	18–012180-145	*Intl*1	*dfr*A19	*qacE*Δ1, *sul*1	-			
Swine	18–012180-261	*Intl*1	*aad*A7	*qac*EΔ1, *sul*1	In142	99.29	981/981	AF234167
Swine	18–012180-267	*Intl*1	-	*qacE*Δ1, *sul*1	-			
Swine	18–012180-282	*Intl*1	*ant*(2″)-Ia, *aad*A2, *aac*(3)-*VIa*	*qac*EΔ1, *sul*1	In293	100	1,531/1,531	DQ520939
Swine	18–012180-381	*Intl*1	-	*qacE*Δ1, *sul*1	-			
Swine	18–012180-398	*Intl*1	-	-	-			
Swine	18–012180-578	*Intl*1	*dfr*A1	*qac*EΔ1, *sul*1	In363	99.57	1,173/1,172	DQ402098
Swine	18–024125-014	*Intl*1	*aad*A24, *aac*(3)-*VIa*	*qac*EΔ1, *sul*1	In288	99.65	10,493/10,484	FJ621588
Swine	18–024125-062	*Intl*1	*aac*(6′)-Ib-cr, *arr*-3, *dfr*A27, *aad*A16	*qac*EΔ1, *sul*1	In1333	99.74	5,296/5,289	CP017059
Swine	18–024127-046	*Intl*1	*dfr*A12, *aad*A2	*qac*EΔ1, *sul*1	-			
Swine	18–024131-069	*Intl*1	*dfr*A12, *aad*A2	*qac*EΔ1, *sul*1	-			
Swine	18–038875-061	*Intl*1	*ant*(2″)-Ia, *cml*A5	*qac*EΔ1, *sul*1	In571	99.97	2,999/2,999	AB285479
Swine	18–038876-010	*Intl*1	-	*qacE*Δ1, *sul*1	-			
Swine	19–020610-021	*Intl*1	*ant*(2″)-Ia, *aad*A2	*qac*EΔ1, *sul*1	In293	100	1,531/1,531	DQ520939
Swine	19–020610-022	*Intl*1	*aad*A1	*qacE*Δ1, *sul*1	-			
Swine	19–020610-039	*Intl*1	-	*qacE*Δ1, *sul*1	-			
Cattle	18–006979-079	*Intl*1	aadA6, *aac*(3)-*VIa*	*qac*EΔ1, *sul*1	-			
Cattle	18–006979-295	*Intl*1	*aad*A12, *ant*(2″)-Ia, *cml*A5	*qac*EΔ1, *sul*1	-			
Cattle	18–012180-049	*Intl*1	*aac*(6′)-Ib4, *aad*A1	-	In48	98.83	1,032/1,029	AF439785
Cattle	18–038877-071	*Intl*1	-	*qac*EΔ1, *sul*1	-			
Poultry	18–006979-061	*Intl*1	*aac*(6′)-Ib4, *aad*A1	-	In48	98.83	1,032/1,029	AF439785
Poultry	18–012180-524	*Intl*1	*aac*(6′)-Ib4, *aad*A1	-	In48	98.83	1,032/1,029	AF439785
Poultry	18–024128-087	*Intl*1	*ant*(2″)-Ia, *aad*A2	*qac*EΔ1, *sul*1	In293	100	1,531/1,531	DQ520939
Poultry	18–038310-008	*Intl*1	*aad*A1, *aac*(3)-*VIa*	-	In790	99.9	1917/1917	JQ326986
Poultry	18–038310-026	*Intl*1	*aac*(6′)-Ib4, *aad*A1	-	In48	98.83	1,032/1,029	AF439785
Poultry	18–038873-015	*Intl*1	*aac*(6′)-Ib4, *aad*A1	-	In48	98.83	1,032/1,029	AF439785
Poultry	18–038873-077	*Intl*1	*aad*A2	*-*	In532	99.57	2,568/2,567	AB121039
Poultry	18–038876-062	*Intl*1	*aac*(6′)-Ib4, *aad*A1	-	In48	98.83	1,032/1,029	AF439785
Poultry	18–038877-021	*Intl*1	*aac*(6′)-Ib4, *aad*A1	-	In48	98.83	1,032/1,029	AF439785
Poultry	19–020610-061	*Intl*1	*aac*(6′)-Ib4, *aad*A1	-	In48	98.83	1,032/1,029	AF439785
Poultry	19–021046-006	*Intl*1	*aac*(6′)-Ib4, *aad*A1	-	In48	98.83	1,032/1,029	AF439785

(-) = not detected

**Table 3:** Correlation of phenotype susceptibility and genotype.

Drug classes	Phenotype: resistant (R)		Phenotype: susceptible (S)						
	Genotype: R	Genotype: S	Genotype: R	Genotype: S	Sensitivity (%)	Specificity (%)	PPV (%)	NPV (%)
Beta-lactams								
AMP	31	3	1	59	96.9	95.2	91.2	98.3
AMC	12	1	2	79	85.7	98.8	92.3	97.5
FOX	13	0	1	80	92.9	100	100	98.8
CRO	18	1	1	74	94.7	98.7	94.7	98.7
Phenicol								
CHL	21	4	0	69	100	94.5	84.0	100
Quinolones								
CIP	15	2	1	76	93.75	97.4	88.2	98.7
NAL	14	1	2	77	87.5	98.7	93.3	97.5
Aminoglycosides								
GEN	27	2	6	59	81.8	96.7	93.1	90.8
STR	39	3	3	49	92.9	94.2	92.9	94.2
Folate pathway inhibitors								
SUL	27	4	3	60	90.0	93.75	87.1	95.2
SXT	16	0	1	77	94.1	100	100	98.7
Tetracycline								
TET	36	4	1	53	97.3	92.9	90.0	98.1
Macrolide								
AZM	6	0	1	87	85.7	100	100	98.9

### Point mutations

Seventeen isolates exhibited decreased resistance to ciprofloxacin (MIC ≥0.12 μg/ml). Fluoroquinolone-resistance genes were identified in 11 of those isolates. The remaining six isolates did not harbor any resistance genes; however, point mutations were detected in these six isolates. Four isolates showed a point mutation in the *gyr*A (D87N) and *par*C (T57S) genes, and the other two isolates had a mutation in only the *par*C (T57S) gene. Five isolates exhibited resistance to nalidixic acid without any specific quinolone resistance gene present, but in four of these five isolates there were point mutations in *gyr*A and *par*C genes and in only the *par*C gene for one isolate. Interestingly, all the isolates showed mutations in the *par*C gene regardless of phenotypic resistance to nalidixic acid or ciprofloxacin.

### Relationship of antimicrobial susceptibility with antimicrobial resistance genes

The association of antimicrobial susceptibility with antimicrobial resistance genes is shown in Table 3. The least discordance among the animal isolates was seen for β-lactams and the most were seen for phenicols.

### Antimicrobial resistance by animal species

**Figure 2:**
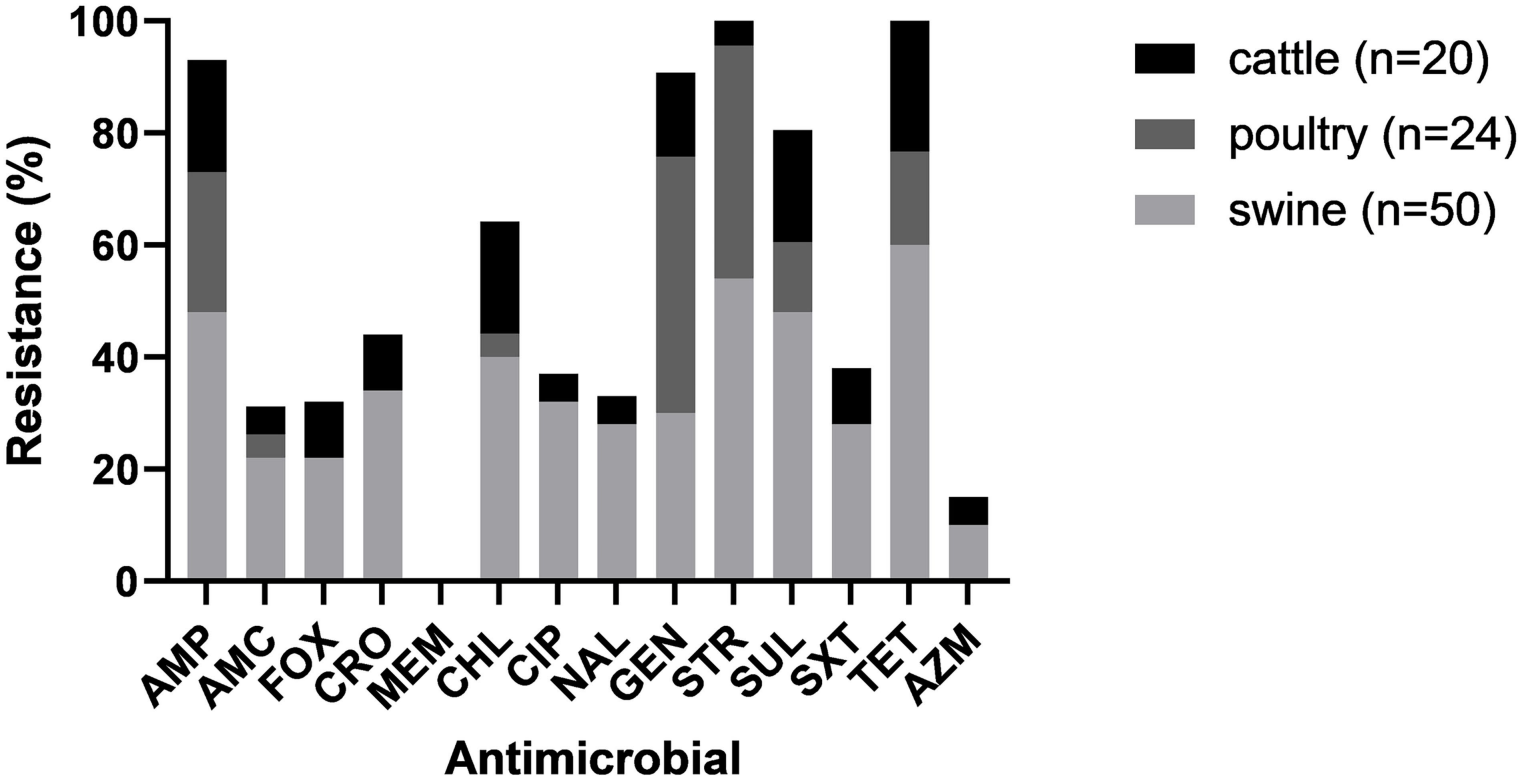
Antimicrobial resistance for each drug among cattle, poultry and swine.

Antimicrobial resistance varied across isolates from different animal species. Isolates from all animal species showed susceptibility to MEM. Isolates from cattle and swine showed resistance to all other antimicrobials in variable frequency. All poultry isolates were susceptible to AMC, FOX, CRO, AZM, CIP and NAL (Figure 2).

The percentage of isolates that were MDR varied among the different animal species. Sixty percent (30/50) of the isolates from swine were MDR; three were resistant to 12 antimicrobials and one was resistant to 13 antimicrobials, being possible XDR. Fewer isolates from cattle (5/24, 16.6%) and poultry (3/24, 12.5%) were MDR.

### Plasmid typing

Antimicrobial resistance genes are often encoded on mobile genetic elements such as plasmids. In this study, 36 plasmid types were identified in 73 (77.7%) isolates. Plasmid profiles differed among each animal species. Thirty-four different plasmids were found among swine isolates, 14 among poultry isolates and 10 among cattle isolates. The most prevalent plasmid was ColRNAI, found in 60.6% of the study isolates: n = 20 (83.3%) in poultry, n = 31 (62%) in swine and n = 6 (30%) in cattle isolates. Other prevalent plasmids were Col440II, present in 35.1% of isolates, Col440I in 24.5% of isolates, RepA1pKPC-CAV1321 in 13.8% of isolates, IncHI2A and IncHI2 in 12.8% of isolates (75% in swine isolates) and others in lower frequency. The presence of the *mcr*-9.1 colistin resistance gene and the presence of the ESBL *bla*_SHV-12_ gene were correlated with the presence of the two plasmids IncHI2A and IncHI2.

### MLST and phylogenetic relationships

Isolates were classified into two distinct sequence types (ST) based on MLST analysis from genome sequences. Ninety-two isolates (97.9%) belonged to ST14 and only two isolates (2.1%) belonged to ST185. Both isolates in ST185 were from cattle. ST14 and ST185 share no common alleles at any of the seven loci that define an allelic profile or ST by MLST analysis.

*S.* Senftenberg is a polyphyletic serovar. Phylogenetic analysis identified two distinct lineages of *S.* Senftenberg in this study that correlated with the MLST results. The smaller clade corresponded to ST185, and the majority of isolates corresponded to ST14 (Supplementary Figure S1).

The addition of closely related representative serotypes from NCBI shows that the two MLST types observed in this study are entirely distinct lineages; and in addition, a third lineage of *S.* Senftenberg which was not observed in this study, becomes visible with isolate NZ_CP007505. This suggests that the serotype designation may not represent a good indicator of the genetic relationships between strains.

The isolates corresponding to ST14 showed a cluster of nine isolates that are significantly divergent from the rest of the isolates. This cluster has accumulated 137 SNPs since sharing a most recent common ancestor with the nearest relatives. Of interest was the presence of common resistance characteristics in this cluster, with eight isolates from poultry and one isolate from cattle showing a similar antimicrobial resistance pattern, resistance genes, class 1 integron, and plasmid profile (Supplementary Figure S2; Table 4). The well differentiated cluster within the ST14 group showed an average distance of 30.8 SNPs (range of 22 to 44 SNP) from a common ancestor.

**Table 4:** Antimicrobial resistance profile, integrons and plasmids in the ST14 differentiated group of 9 isolates.

ID isolate	Source	State	Year	Antimicrobial Susceptibility test	Antimicrobial resistance genes	Integrons	Plasmids
19–021046-006	Poultry	IA	2017	AMP, GEN, STR	*bla*_TEM-1_, *aac*(6′)-Ib4, *aad*A1, *acc*(6′)-laa	*Intl*1, *aac*(6′)-Ib4, *aad*A1	Col440II, ColRNAI, IncFIB, IncFII.
18–006979-061	Poultry	MO	2014	AMP, GEN, STR	*bla*_TEM-1_, *aac*(6′)-Ib4, *aad*A1, *acc*(6′)-laa	*Intl*1, *aac*(6′)-Ib4, *aad*A1	Col440II, Col440I, ColRNAI, IncFIB, IncFII.
18–038873-015	Poultry	MO	2016	GEN, STR	*aac*(6′)-Ib4, *aad*A1, *acc*(6′)-laa	*Intl*1, *aac*(6′)-Ib4, *aad*A1	Col440II, ColRNAI, IncFIB, IncFII.
18–038877-021	Poultry	IA	2017	AMP, GEN, STR	*bla*_TEM-1_, *aac*(6′)-Ib4, *aad*A1, *acc*(6′)-laa	*Intl*1, *aac*(6′)-Ib4, *aad*A1	Col440II, ColRNAI, IncFIB, IncFII.
18–024128-065	Poultry	IN	2016	GEN	*acc*(6′)-laa	Not found	Col440II, ColRNAI
18–038310-026	Poultry	IA	2016	GEN, STR	*aac*(6′)-Ib4, *aad*A1	*Intl*1, *aac*(6′)-Ib4, *aad*A1	Col440II, ColRNAI, IncFIB, IncFII.
19–020610-061	Poultry	AR	2017	AMP, GEN, STR	*bla*_TEM-1_, *aac*(6′)-Ib4, *aad*A1, *acc*(6′)-laa	*Intl*1, *aac*(6′)-Ib4, *aad*A1	Col440II, ColRNAI, IncFIB, IncFII.
18–012180-524	Poultry	MO	2015	AMP, AMC, GEN	*bla*_TEM-1_, *aac*(6′)-Ib4, *aad*A1, *acc*(6′)-laa	*Intl*1, *aac*(6′)-Ib4, *aad*A1	Col440II, ColRNAI, IncFIB, IncFII.
18–012180-049	Cattle	IN	2015	AMP, GEN, STR	*bla*_TEM-1_, *aac*(6′)-Ib4, *aad*A1, *acc*(6′)-laa	*Intl*1, *aac*(6′)-Ib4, *aad*A1	Col440II, Col440I, ColpVC

## Discussion

A previous study of animal and human isolates in the USA (Stepan et al., 2011) showed that human strains of *S.* Senftenberg were susceptible to all of the antimicrobials tested, whereas the animal isolates showed a range of resistance, with most isolates being resistant to two or more antimicrobials.

In this study, isolates from swine and cattle showed resistance to 13 antimicrobials in different frequencies, whereas poultry isolates showed resistance to only six antimicrobials tested; these results differ from Stepan et al. (Stepan et al., 2011) where the rate of resistance to antimicrobials was similar across the host species (swine, cattle and poultry). Other important findings of our study were the presence of three isolates from swine resistant to 12 antibiotics and one resistant to 13 antibiotics.

In this study we found two sequence types associated with *S.* Senftenberg (ST14 = 97.8% and ST185 = 2.1%), whereas Stepan et al. (Stepan et al., 2011) found three sequence types (ST14 = 85.7%, ST185 = 13.2% and ST145 = 1%), with ST145 only found in one isolate from swine. The two lineages identified in our study and the branches within the lineage corresponding to ST14 did not show host specificity. This information, combined with the frequent isolation of this serotype from feed, may indicate that the serotype is more likely to be introduced from a common external source rather than circulating long-term within animal populations. In a study in China, El Ghany et al. (El Ghany et al., 2016) identified two phylogenetically distinct clades of *S*. Senftenberg by SNP analysis. Variations were in the *Salmonella* pathogenicity island (SPI)-1 and SPI-2 that exhibited distinct biochemical and phenotypic signatures. Clade 1 isolates comprised three sequence types: ST185, ST217, and ST1751, being single or double locus variants relative to one another. In contrast, clade 2 isolates included only ST14. In our study, the two distinct lineages also differ in the ST. Even though there are other STs observed in other datasets, these STs are single or double locus variants relative to one another.

We observed that there are other serotypes that fall between the two lineages, indicating that they are two completely independent lineages in *S.* Senftenberg and not a single serotype that diverged over time.

As may be expected based on the phylogenetic diversity, resistance patterns among *S.* Senftenberg isolates differ significantly among isolates. In our study, the most common resistance profile was AMP, GEN, STR (n = 5 isolates), whereas Stepan et al. (Stepan et al., 2011) found STR, TET, SXT the most common (n = 4 isolates).

In Veeraraghavan et al. study (Veeraraghavan et al., 2019) beta-lactam resistance was associated with the presence of the *bla*_TEM-1_, *bla*_OXA-9_, *bla*_CMY-2_, and *bla*_NDM-1_ genes, resistance to aminoglycosides was associated with five genes, namely *aac*(6′)-Ia, *aac*(6′)-Ib, *aph*(3′′)-Ib, *aph*(6′)-Ib and *ant*(2′′)-Ia, and sulfonamide resistance was associated with the *sul*1 and *sul*2 genes and resistance to chloramphenicol with the *flor*R gene. In our study, we found *bla*_TEM-1_, *bla*_CMY-2_ and *bla*_SHV-12_. The *bla*_SHV-12_ gene, an ESBL that shows resistance to ceftriaxone, was found in five isolates from swine. These five isolates also carried other antimicrobial genes against beta-lactams, aminoglycosides, sulfonamides, tetracyclines and rifamycin: *bla*_TEM-1_, *aac*(3)-II, *aac*(6’)-IIc, *aac*(6’)-Ib, *sul*1, *sul*2, *tet*D, *arr*-269927220, and all of them were positive for IncHI2A, IncHI2, RepA pKPC-CAV1321 plasmids. IncHI2 plasmid replicon has been reported to encode *bla*_SHV-12_, the most predominant ESBL within *Enterobacteriaceae* (Liakopoulos et al., 2018), and can be transfer among diverse bacterial population. Expanded monitoring of *Salmonella* from swine for this gene would be appropriate to evaluate the extent of the gene in the U.S. swine population to determine if swine are a significant reservoir of ceftriaxone-resistance. In general, both *aph*(3′′)-Ib and *aph*(6)-Id genes were predominantly found in streptomycin-resistant isolates in other MDR *S. enterica* (Cohen et al., 2020) and we found them in 29.8% isolates. Additionally, these 2 genes are found together in an HI type plasmid (McMillan et al., 2019). The *acc*(6′)-Iaa gene that confers resistance to tobramycin, kanamycin and amikacin, and that was found in all our isolates, was previously found in *S*. Typhimurium and *S*. Infantis; but it seems to have no clinical significance and evolutionary advantage (Jovčić et al., 2020). In our study *sul*1 and *sul*2 genes were also associated with sulfonamide resistance, and *flo*R gene the most prevalent associated with chloramphenicol resistance, although other genes also were found.

In an XDR *S*. Senftenberg isolate from a human clinical case in India (Veeraraghavan et al., 2019), fluoroquinolone resistance was attributed to substitutions in the *gyr*A (S83Y, D87G) and *par*C (S80I) genes. The *par*C substitution appears to be a characteristic mutation present in quinolone-resistant *S.* Senftenberg isolates from human cases (Whichard et al., 2007). In the Wichard study (Whichard et al., 2007), all *S.* Senftenberg isolates had *par*C mutations (T57S and S80I). We also found *par*C mutations in all isolates; but in contrast to the human isolates, we found S83S and D87N mutations in *gyr*A, and only the T57S mutation in *par*C in the animal isolates. The widespread presence of *par*C mutations without corresponding resistance does not appear to be specific to *S.* Senftenberg strains. One study of serotype Paratyphi (Qian et al., 2020) showed *par*C mutations in all isolates (n = 8) with 7 isolates being susceptible to ciprofloxacin. This clearly illustrates that the presence of mutations does not necessarily correspond to phenotypic resistance (Sáenz et al., 2003). On the other hand, one study in nontyphoidal *S. enterica* isolated from pigs in Thailand showed resistance to fluoroquinolones, either in the presence or absence of genes and/or mutations, and *par*C mutation (T57) was found in 62.4% of the isolates (Poomchuchit et al., 2021).

When we compared our *S.* Senftenberg results with a study that evaluated antimicrobial susceptibility patterns found in other serovars isolated from poultry (Cohen et al., 2020), the percentage of MDR in *S*. Senftenberg from poultry (12.5%) was higher than *S.* Orion (10%) and lower than *S.* Kentucky (97.4%), *S.* Hadar (80%), *S.* Java (75%), *S.* Infantis (60%), *S.* Bredeney (50%), *S.* Montevideo (40%), *S.* Newport (30%), *S.* Virchow (30%), *S.* Blockeley (27.3%), *S*. Muenchen (25%). In MDR swine *Salmonella* isolates, Argüello et al. (Argüello et al., 2018) demonstrated the importance of class 1 integrons and certain genes. In this study, 60% (n = 30) of isolates from swine origin showed MDR, and in 70% (n = 21) of these isolates we observed the presence of a class 1 integron.

The number of plasmids did not correlate with MDR or XDR, as we found isolates with as many as seven or eight plasmids that were resistant to only one or two antibiotics and isolates with only one plasmid with resistance to seven antibiotics.

Of the other genes found in *S.* Senftenberg strains, the presence of the *mcr*-9 gene that confers resistance to colistin was of interest. This is a novel *mcr* homologue detected in MDR colistin-susceptible *Salmonella* Typhimurium isolated from a patient in the USA in 2010 (Carroll et al., 2019). The *mcr*-9 gene was shown to be capable of conferring phenotypic resistance to colistin in numerous genera of *Enterobacteriaceae*, and it is harbored in IncHI2 and/or IncHI2A replicons (Carroll et al., 2019). The Sensititre CMV4AGNF plate did not include colistin, so we were unable to determine if this gene was expressed in the *S.* Senftenberg isolates. However, a set of 57 *Salmonella* isolates, including four *S*. Senftenberg swine isolates used in the current study, were positive for *mcr*-9 when tested using the Sensititre GNX3F plate that contains colistin (unpublished data). Of the 57 isolates, only one had an MIC of 2 ng/μl (resistant), and the remaining 56 had an MIC of 1 ng/μl or lower (susceptible), with the four isolates from this current study having MIC values equal to or lower than 0.5 ng/μl. These results agree with the report of Tyson et al. (Tyson et al., 2020) affirming that the *mcr*-9 gene in *Salmonella* is not associated with colistin resistance in the USA. All isolates positive for this gene in our study carried two plasmids (IncHI2A and IncHI2), while all other isolates were negative for these two plasmids, so we could associate the presence of these plasmids with the presence of the *mcr-*9 gene. In other studies, the *mcr-*1 gene was found in IncHI2/ST3, IncI2, and IncX4 plasmids in isolates from animals and humans (Stefaniuk and Tyski, 2019).

In addition to antimicrobial resistance genes, antiseptic resistance genes are important because disinfectants are used in farm environments. Benzalkonium chloride is a surface-active QAC (quaternary ammonium compound), and it is used as a farm disinfectant. The *qac*EΔ1 is frequently present in *E. coli* and other enteric bacteria (Zou et al., 2014). In this study 25 isolates carried the *qac*EΔ1 gene that has been identified in mobile genetic elements. The *qac*E and *qac*EΔ1 genes are located on an integron, a *qac*EΔ1 represents a disrupted form of *qac*E that evolved as a result of the insertion of a DNA segment near the 3′ end of the *qac*E gene carrying a *sul*1 sulfonamide resistance determinant (Paulsen et al., 1993). All but one of the *S.* Senftenberg isolates positive for *qac*EΔ1 gene carried the *sul*1 gene. The one exception carried the *sul*2 and *sul*3 genes. We also detected the presence of class 1 integrons in the *qac*EΔ1 positive isolates. Class 1 integrons are associated with an *Intl*1 integrase in the 5′ conserved sequence (CS) and with a 3′ CS conferring resistance to antibiotics (sulfonamides) and bactericidal compounds (quaternary ammonium) of the integron (Deng et al., 2015). Class 1 integrons have previously been detected in *S*. Senftenberg (Vo et al., 2006), but to our knowledge, there are no reports of class 2 or class 3 integrons in this serovar. We found 10 isolates harboring a class 1 integron homologous to the In48 integron (98.83%) that carries the aminoglycoside 6’-N-acetyltransferase (*aac*A4) gene. In our isolates, this integron carried the aminoglycoside N-acetyltransferase *aac*(6′)-Ib4 gene (*aac*(6′)-Ib allele) and the *aad*A1 gene. The frequent use of QAC may facilitate resistance to disinfectants, and QACs may serve as important selective agents in MDR pathogens (Sinwat et al., 2021). In the present study, all *S.* Senftenberg isolates carrying the *qac*EΔ1 gene were MDR.

Concordance between the presence of antimicrobial resistance genes and phenotypic resistance profiles were seen in 98.1% of isolates for the antimicrobials tested. The presence of resistance genes do not necessarily confer phenotypic resistance, and occasionally, an isolate will display antimicrobial resistance without the presence of a known resistance gene (Piddock, 2016). The presence or absence of resistance genes is not enough for the phenomenon of antimicrobial resistance. Mechanisms such as enzyme activation, target modification or protection, regulation of gene expression, or changes in the cell wall can play an important role in the resistance of antimicrobials (Paudyal et al., 2019).

## Conclusion

This study provided an analysis of retrospective data of *Salmonella* Senftenberg and information about the antimicrobial susceptibility and genomic characteristics in diagnostic isolates of *S.* Senftenberg from production animals in the USA. This study reports the genotype-phenotype homogeneity and variability of *S.* Senftenberg of animal origin. The ability of *S.* Senftenberg to persist in the environment, to cause disease in various animal species, to be a potential risk of transmission to humans and to harbor resistance to critical antimicrobial and mobile elements capable of dissemination of acquired resistance genes makes *S.* Senftenberg an important public health pathogen. In this study we found that 39.4% of the isolates tested displayed multidrug resistance, and four isolates were potentially extensively drug resistant. This has important implications for both animal and human health, due to possible transmission of MDR bacteria from animal to animal or animal to human (zoonotic), and the difficulty in treatment of resistant bacteria. These data highlight the need to strengthen surveillance to detect the prevalence and transmission of nontyphoidal *Salmonella* species because of the emergence of MDR strains. It is critical to identify the emergence of these strains as early as possible to avoid further dissemination and establish control procedures. This data is useful for future studies on *S.* Senftenberg and to further understand this pathogen as few studies exist on the antimicrobial susceptibility, genotypic profiles and genetic diversity of *Salmonella* Senftenberg of animal origin.

## Data availability statement

The datasets presented in this study can be found in online repositories. The names of the repository/repositories and accession number(s) can be found at: https://www.ncbi.nlm.nih.gov/, bioproject/PRJNA785813.

## Author contributions

MS and LS: conceptualization. MS and JH: methodology, formal analysis, and visualization. JH: software, formal analysis and validation. MS: investigation and writing – original draft preparation. LS: resources, supervision, project administration and funding acquisition. MS, JH, BM, TM and LS: writing – review and editing. LS: Supervision. All authors contributed to the article and approved the submitted version.

## Funding

This project was supported in part by an appointment to the Research Participation Program at the Animal and Plant Health Inspection Service, United States Department of Agriculture, administered by the Oak Ridge Institute for Science and Education through an interagency agreement between the US Department of Energy and USDA APHIS.

## Conflict of interest

The authors declare that the research was conducted in the absence of any commercial or financial relationships that could be construed as a potential conflict of interest.

## Publisher’s note

All claims expressed in this article are solely those of the authors and do not necessarily represent those of their affiliated organizations, or those of the publisher, the editors and the reviewers. Any product that may be evaluated in this article, or claim that may be made by its manufacturer, is not guaranteed or endorsed by the publisher.

## Footnotes

^1^ https://github.com/seeman/abricate/

^2^ https://github.com/USDA-VS/vSNP
