# Supplementary material for: Antimicrobial resistance and genomic characterization of Salmonella enterica serovar Senftenberg isolates in production animals from the United States
Source: Front Microbiol. 2022 Nov 3;13:979790. doi: 10.3389/fmicb.2022.979790 (PMC9668867; doi:10.3389/fmicb.2022.979790)
Supplement: Supplementary file 1 [file Data_Sheet_1.docx]

**Supplementary Table 1.** Phenotypic resistance profiles and the resistance genes present in *Salmonella* Senftenberg isolates displaying antimicrobial resistance.

|  | **year** | **source** | **ID** | **Antimicrobial Susceptibility** | **Antimicrobial Resistance genes** | **Biocide Resistance gene** | **Class 1 Integron** | **MDR** |
| --- | --- | --- | --- | --- | --- | --- | --- | --- |
|  | 2014 | Swine | 18-006979-039 | AMP AMC FOX CRO | *bla*CMY-2, *aac*(6')-Iaa |  |  | + |
|  | 2014 | Swine | 18-006979-062 | AMP CHL CIP NAL STR SUL SXT TET | *bla*TEM-1, *cat*A2, *flo*R, *qnr*B2, *aac*(6')-Ib, *qnr*B2, *aad*A2, *aph*(3'')-Ib, *aph*(6)-Id, *sul*1, *sul*2, *tet*(D), *dfr*A19, *aph*(3')-Ia, *aac*(6')-Iaa, *mcr*-9.1 | *qac*EΔ1 | + | + |
|  | 2014 | Swine | 18-006979-088 | TET | *tet*(B), *aac*(6')-Iaa |  |  |  |
|  | 2014 | Swine | 18-006979-089 | AMP CRO CHL CIP GEN SUL TET | *aph*(3')-Ia, *aac*(6')-Iaa |  |  | + |
|  | 2014 | Swine | 18-006979-143 | AMP CRO CHL CIP NAL GEN STR SUL SXT TET | *bla*SHV-12, *bla*TEM-1, *cat*A2, *flo*R, *qnr*B2, *aac*(3)-II, *aac*(6')-IIc, *aac*(6')-Ib, *aad*A2, *aph*(3'')-Ib, *aph*(6)-Id, *sul*1, *sul*2, *tet*(D), *dfr*A19, *aph*(3')-Ia, *aac*(6')-Iaa, *ere*A, *mcr*-9.1, *arr*-269927220 | *qac*EΔ1 | + | + |
| * | 2014 | Swine | 18-006979-166 | AMP AMC FOX CRO CHL CIP NAL GEN STR SUL SXT TET | *bla*CMY-2, *bla*TEM-1, *cml*A5, *flo*R, *ant*(2'')-Ia, *aph*(3'')-Ib, *aph*(6)-Id, *sul*1, *sul*2, *tet*(A), *dfr*A1, *aph*(3')-Ia, *aac*(6')-Iaa, *gyr*A(D87N) | *qac*EΔ1 | + | + |
|  | 2014 | Swine | 18-006979-167 | AMP AMC FOX CRO STR SUL TET | *bla*CMY-2, *aad*A1, *aph*(3'')-Ib, *aph*(6)-Id, *sul*1, *tet*(A), *aph*(3')-Ia, *aac*(6')-Iaa | *qac*EΔ1 | + | + |
|  | 2014 | Swine | 18-006979-173 | AMP CRO CHL CIP GEN STR SUL TET | *bla*SHV-12, *bla*TEM-1, *cat*A2, *qnr*B2, *aac*(3)-II, *aac*(6')-IIc, *aac*(6')-Ib, *aad*A2, *sul*1, *sul*2, *tet*(D), *aac*(6')-Iaa, *arr*-269927220 | *qac*EΔ1 | + | + |
|  | 2014 | Swine | 18-006979-178 | SUL TET AZT | *erm*(42), *sul*2, *tet*(A), *aac*(6')-Iaa |  |  | + |
|  | 2014 | Swine | 18-006979-242 | GEN STR | *aac*(3)-Iva, *aph*(3'')-Ib, *aph*(6)-Id, *aph*(4)-Ia, *aac*(6')-Iaa |  |  |  |
|  | 2014 | Swine | 18-006979-306 | AMP CHL TET | *bla*TEM-150, *aac*(6')-Iaa, *flo*R, *tet*(A) |  |  | + |
|  | 2014 | Swine | 18-006979-312 | AMP GEN STR TET | *bla*TEM-1, *aac*(3)-Iva, *aph*(3'')-Ib, *aph*(6)-Id, *tet*(B), *aph*(3')-Ia, *aph*(4)-Ia, *aac*(6')-Iaa, *ble*O |  |  | + |
|  | 2014 | Swine | 18-006979-346 | CIP NAL | *aac*(6')-Iaa |  |  |  |
|  | 2014 | Swine | 18-006979-395 | STR | *aac*(6')-Iaa |  |  |  |
|  | 2014 | Swine | 18-006979-483 | AMP CIP NAL STR TET | *bla*TEM-1, *qnr*B19, *aph*(3'')-Ib, *aph*(6)-Id, *tet*(B), *aac*(6')-Iaa |  |  | + |
|  | 2014 | Swine | 18-006979-525 | AMP CHL STR SUL TET | *aac*(6')-Iaa |  |  | + |
|  | 2015 | Swine | 18-012180-030 | CIP NAL GEN STR TET | *qnr*B77, *aac*(3)-Via, *aad*A1, *aph*(3'')-Ib, *aph*(6)-Id, *tet*(A), *dfr*A15, *aac*(6')-Iaa |  | + | + |
|  | 2015 | Swine | 18-012180-144 | AMP AMC FOX CRO | *bla*CMY-2, *aac*(6')-Iaa |  |  | + |
|  | 2015 | Swine | 18-012180-145 | AMP CRO CHL GEN STR SUL SXT TET | *bla*SHV-12, *bla*TEM-1, *cat*A2, *aac*(3)-II, *aac*(6')-IIc, *aac*(6')-Ib, *aad*A2, *aph*(3'')-Ib, *aph*(6)-Id, *sul*1, *sul*2, *tet*(D), *dfr*A19, *aph*(3')-Ia, *aac*(6')-Iaa, *ere*A, *mcr*-9.1, *arr-*269927220 | *qac*EΔ1 | + | + |
|  | 2015 | Swine | 18-012180-261 | GEN STR SUL SXT TET AZT | *arm*A, *aad*A5, *aad*A7, *sul*1, *dfr*A1, *mph*(E),*msr*(E), *aph*(3')-Ia, *aac*(6')-Iaa | *qac*EΔ1 | + | + |
|  | 2015 | Swine | 18-012180-267 | STR SUL TET | *aad*A1, *aph*(3'')-Ib, *aph*(6)-Id, *sul*1, *tet*(A), *aac*(6')-Iaa | *qac*EΔ1 | + | + |
| * | 2015 | Swine | 18-012180-282 | AMP AMC FOX CRO CHL CIP NAL GEN STR SUL SXT TET AZT | *bla*CMY-2, *bla*TEM-1, *flo*R, *aac*(3)-VIa, *ant*(2'')-Ia, *aad*A2, *aad*A6, *aph*(3'')-Ib, *aph*(6)-Id, *sul*1, *sul*2, *tet*(A), *tet*(M), *dfr*A34, *mph*(A), *aph*(3')-Ia, *aac*(6')-Iaa, *gyr*A(D87N) | *qac*EΔ1 | + | + |
|  | 2015 | Swine | 18-012180-381 | AMP CHL CIP NAL GEN STR SUL SXT TET | *bla*TEM-1, *cat*A2, *qnr*B2, *aac*(3)-II, *aac*(6’)-IIc, *aac*(6')-Ib, *aad*A2, *aph*(3'')-Ib, *aph*(6)-Id, *sul*1, *sul*2, *tet*(D), *dfr*A19, *aph*(3')-Ia, *aac*(6')-Iaa, *ere*A, *mcr*-9.1, *arr*-269927220 | *qac*EΔ1 | + | + |
|  | 2015 | Swine | 18-012180-398 | AMP AMC FOX CRO CHL CIP NAL STR SUL TET | *bla*CMY-2, *bla*TEM-1, *flo*R, *aph*(3'')-Ib, *aph*(6)-Id, *sul*2, *tet*(A), *tet*(M), *aph*(3')-Ia, *aac*(6')-Iaa, *ble*O, *gyr*A(D87N) |  | + | + |
|  | 2015 | Swine | 18-012180-578 | AMP AMC FOX CRO CHL GEN STR SUL SXT TET | *bla*CMY-2, *bla*TEM-1, *flo*R, *aac*(3)-IVa, *aph*(3'')-Ib, *aph*(6)-Id, *sul*1, *sul*2, *tet*(A), *dfr*A1, *aph*(3')-Ia, *aac*(6')-Iaa, *aph*(4)-Ia, *ble*O | *qac*EΔ1 | + | + |
|  | 2016 | Swine | 18-024125-008 | AMP CHL STR SUL TET | *aac*(6')-Iaa |  |  | + |
|  | 2016 | Swine | 18-024125-014 | STR TET | *bla*CMY-2, *aac*(3)-VIa, *aad*A24, *aph*(3'')-Ib, *aph*(6)-Id, *sul*1, *tet*(A), *aac*(6')-Iaa | *qac*EΔ1 | + |  |
|  | 2016 | Swine | 18-024125-062 | CHL CIP NAL SUL SXT TET | *aac*(6')-Ib-cr, *qnr*B6, *aad*A16, *aph*(6)-Ic, *sul*1, *tet*(A), *dfr*A27, *aph*(3')-IIa, *aac*(6')-Iaa, *arr-*3, *ble*Tn5 | *qac*EΔ1 | + | + |
| * | 2016 | Swine | 18-024127-046 | AMP AMC FOX CRO CHL CIP NAL STR SUL SXT TET AZT | *bla*CMY-2, *mph*(A), *flo*R, *qnr*B19, *aad*A2, *aph*(3'')-Ib, *aph*(6)-Id, *sul*1, *sul*2, *tet*(A), *tet*(M), *dfr*A12, *aph*(3')-Ia, *aac*(6')-Iaa | *qac*EΔ1 | + | + |
|  | 2016 | Swine | 18-024131-069 | AMP AMC FOX CRO CHL STR SUL SXT TET | *bla*CMY-2, *flo*R, aadA2, aph(3'')-Ib, aph(6)-Id, sul1, sul2, tet(A), dfrA12, aph(3')-Ia, aac(6')-Iaa | *qac*EΔ1 | + | + |
| * | 2017 | Swine | 18-038875-061 | AMP AMC FOX CRO CHL CIP NAL GEN STR SUL SXT TET | *bla*CMY-2, *bla*TEM-1, *erm*(B), *cml*A5, *flo*R, *qnr*B77, *ant*(2'')-Ia, *acc*(3'')-VIa, *aph*(3'')-Ib, *aph*(6)-Id, *sul*1, *sul*2, *tet*(A), *tet*(X), *dfr*A15, *aac*(6')-Iaa | *qac*EΔ1 | + | + |
|  | 2017 | Swine | 18-038876-010 | AMP CRO CHL GEN STR SUL TET | *bla*SHV-12, *bla*TEM-1, *cat*A2, *aac*(3)-II, *aac*(6’)-IIc, *aac*(6')-Ib, *aad*A2, *sul*1, *sul*2, *tet*(D), *aph*(3')-Ia, *aac*(6')-Iaa, *ere*A, *arr*-269927220 | *qac*EΔ1 | + | + |
|  | 2017 | Swine | 18-038877-075 | CIP NAL | *qnr*B19, *aac*(3)-Iva, *aac*(6')-Iaa |  |  |  |
|  | 2017 | Swine | 19-020610-021 | AMP AMC FOX CRO CHL STR SUL SXT TET AZT | *bla*TEM-1, *bla*CMY-2, *mph*(A), *flo*R, *aac*(3)-VIa, *aac*(3)-VIa, *ant*(2'')-Ia, *aad*A2, *aad*A6, *aph*(3'')-Ib, *aph*(6)-Id, *sul*1, *sul*2, *tet*(A), *tet*(M), *dfr*A34, *aph*(3')-Ia, *aac*(6')-Iaa | *qac*EΔ1 | + | + |
|  | 2017 | Swine | 19-020610-022 | STR SUL TET | *aad*A1, *aph*(3'')-Ib, *aph*(6)-Id, *sul*1, *tet*(A), *aac*(6')-Iaa | *qac*EΔ1 | + | + |
|  | 2017 | Swine | 19-020610-039 | AMP CRO CHL CIP NAL GEN STR SUL SXT TET | *bla*SHV-12, *bla*TEM-1, *cat*A2, *qnr*B2, *aac*(3)-II, *aac*(6')-IIc, *aac*(6')-Ib, *aad*A2, *aph*(3'')-Ib, *aph*(6)-Id, *sul*1, *sul*2, *tet*(D), *dfr*A19, *ere*A, *aph*(3')-la, *aac*(6')-Iaa, *mcr*-9.1, *arr*-269927220 | *qac*EΔ1 | + | + |
|  | 2014 | Cattle | 18-006979-079 | AMP FOX CRO CHL CIP NAL STR SUL SXT TET | *bla*TEM-1, *bla*CMY-2, *flo*R, *aac*(3)-VIa, *aad*A6, *aph*(3'')-Ib, *aph*(6)-Id, *sul*1, *sul*2, *tet*(A), *tet*(B), *tet*(M), *dfr*A34, *aac*(6')-Iaa, *gyr*A(D87N) | *qac*EΔ1 | + | + |
|  | 2014 | Cattle | 18-006979-094 | TET | *tet*(B), aac(6')-Iaa |  |  |  |
|  | 2014 | Cattle | 18-006979-295 | AMP AMC FOX CRO CHL GEN STR SUL TET | *bla*TEM-1, *bla*CMY-2, *cml*A5, *ant*(2'')-Ia, *aad*A12, *aph*(3'')-Ib, *aph*(6)-Id, *sul*1, *sul*2, *tet*(A), *aac*(6')-Iaa | *qac*EΔ1 | + | + |
|  | 2014 | Cattle | 18-006979-463 | TET AZT | *erm*(42), tet(B), aac(6')-Iaa |  |  |  |
|  | 2015 | Cattle | 18-012180-049 | AMP GEN STR | *bla*TEM-1, aac(6')-Ib4, aadA1, aac(6')-Iaa |  | + |  |
|  | 2016 | Cattle | 18-024127-044 | CHL STR SUL TET | floR, aph(3'')-Ib, aph(6)-Id, sul2, tet(A), aac(6')-Iaa |  |  | + |
|  | 2017 | Cattle | 18-038877-071 | AMP CHL GEN STR SUL SXT TET | *bla*TEM-1, *cat*A2, *qnr*B2, *aac*(3)-II, *aac*(6')-IIc, *aad*A2, *aph*(3'')-Ib, *aph*(6)-Id, *sul*1, *sul*2, *tet*(D), *dfr*A19, *aph*(3')-Ia, *aac*(6')-Iaa, *ere*A, *mcr*-9.1, *arr*-269927220 | *qac*EΔ1 | + | + |
|  | 2014 | Poultry | 18-006979-061 | AMP GEN STR | *bla*TEM-1, aac(6')-Ib4, aadA1, aac(6')-Iaa |  | + |  |
|  | 2015 | Poultry | 18-012180-442 | SUL | *aac*(6')-Iaa |  |  |  |
|  | 2015 | Poultry | 18-012180-524 | AMP AMC GEN | *bla*TEM-1, *aac*(6')-Ib4, *aac*(6')-Iaa |  | + | + |
|  | 2016 | Poultry | 18-024128-065 | GEN | *aac*(6')-Iaa |  |  |  |
|  | 2016 | Poultry | 18-024128-087 | AMP GEN STR SUL TET | *bla*TEM-1, *ant*(2'')-Ia, *aad*A1, *aad*A2, *aph*(3'')-Ib, *aph*(6)-Id, *sul*1, *tet*(A), *aph*(3')-Ia, *aac*(6')-Iaa | *qac*EΔ1 | + | + |
|  | 2016 | Poultry | 18-038310-008 | GEN STR | *aac*(3)-Via, *aad*A1, *aac*(6')-Iaa |  | + |  |
|  | 2016 | Poultry | 18-038310-026 | GEN STR | *aac*(6')-Ib4, *aad*A1, *aac*(6')-Iaa |  | + |  |
|  | 2016 | Poultry | 18-038873-015 | GEN STR | *aac*(6')-Ib4, *aad*A1, *aac*(6')-Iaa |  | + |  |
|  | 2016 | Poultry | 18-038873-077 | CHL GEN STR SUL TET | *cml*A1, floR, *ant*(2'')-Ia, *aad*A1, *aad*A2, *aph*(3'')-Ib, *aph*(6)-Id, *sul*2, *sul*3, *tet*(A), *aac*(6')-Iaa | *qac*EΔ1 | + | + |
|  | 2017 | Poultry | 18-038876-062 | STR TET | *aac*(6')-Ib4, *aad*A1, *tet*(B), *aac*(6')-Iaa, *mcr*-9.1 |  | + |  |
|  | 2017 | Poultry | 18-038877-021 | AMP GEN STR | *bla*TEM-1, *aac*(6')-Ib4, *aad*A1, *aac*(6')-Iaa |  | + |  |
|  | 2017 | Poultry | 18-038877-047 | TET | *tet*(B), *mcr*-9.1, *aac*(6')-Iaa |  |  |  |
|  | 2017 | Poultry | 19-020610-061 | AMP GEN STR | *bla*TEM-1, *aac*(6')-Ib4, *aad*A1, *aac*(6')-Iaa |  | + |  |
|  | 2017 | Poultry | 19-021046-006 | AMP GEN STR | *bla*TEM-1, *aac*(6')-Ib4, *aad*A1, *aac*(6')-Iaa |  | + |  |

*Potentially extensively drug-resistant. MDR= multidrug resistance.

**
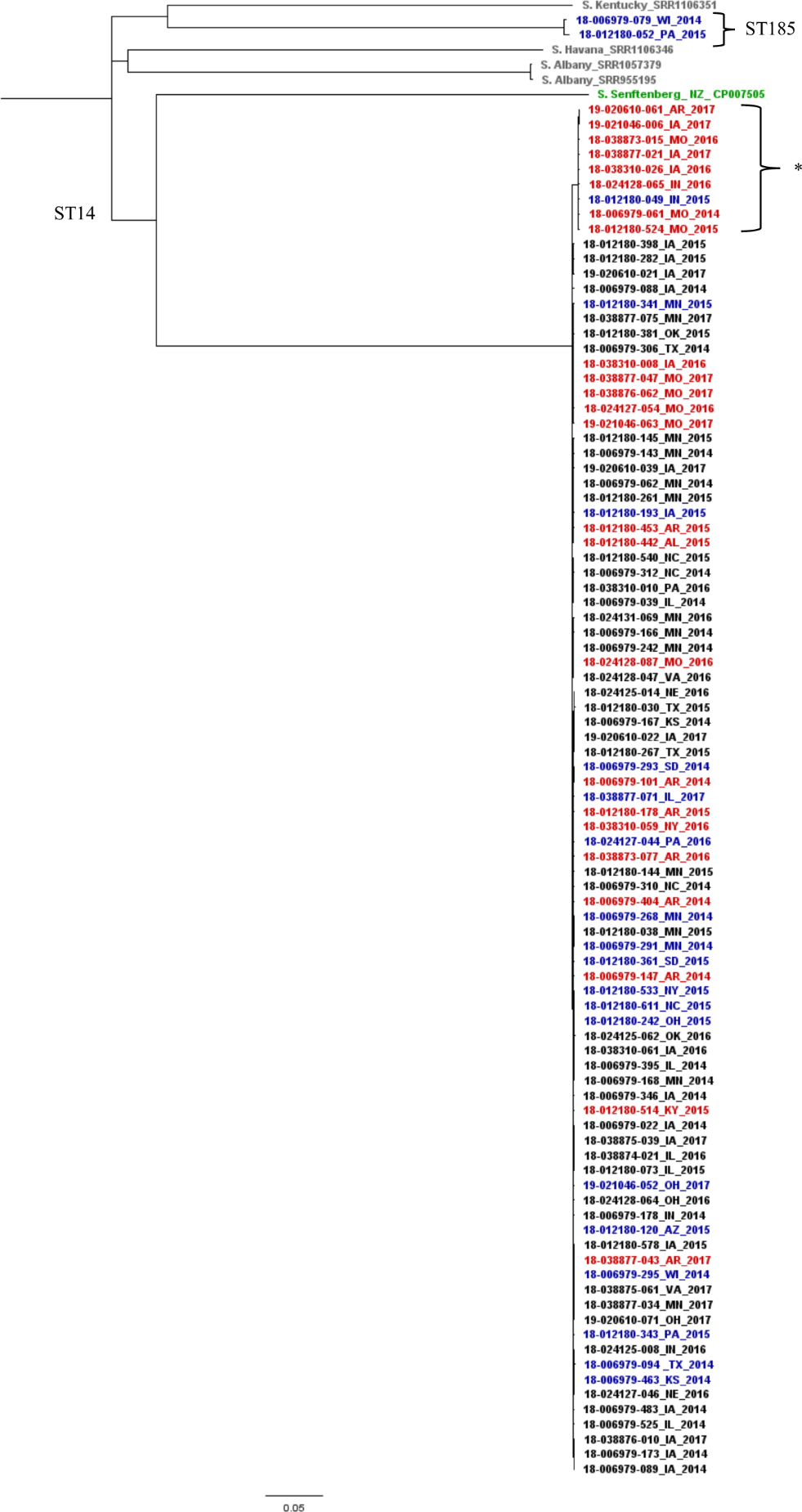
**

**Supplementary Figure 1.** Phylogenetic 0.8 majority kSNP tree of *Salmonella* Senftenberg isolates. Isolates from swine are in black, isolates from poultry in red, and isolates from cattle are in blue. The *S.* Senftenberg reference strain is in green. With the addition of other *Salmonella* serotypes (in grey) we can observe the polyphyletic nature of the *S.* Senftenberg serotype. A distinct group is shown inside the *S.* Senftenberg ST14 group.

**
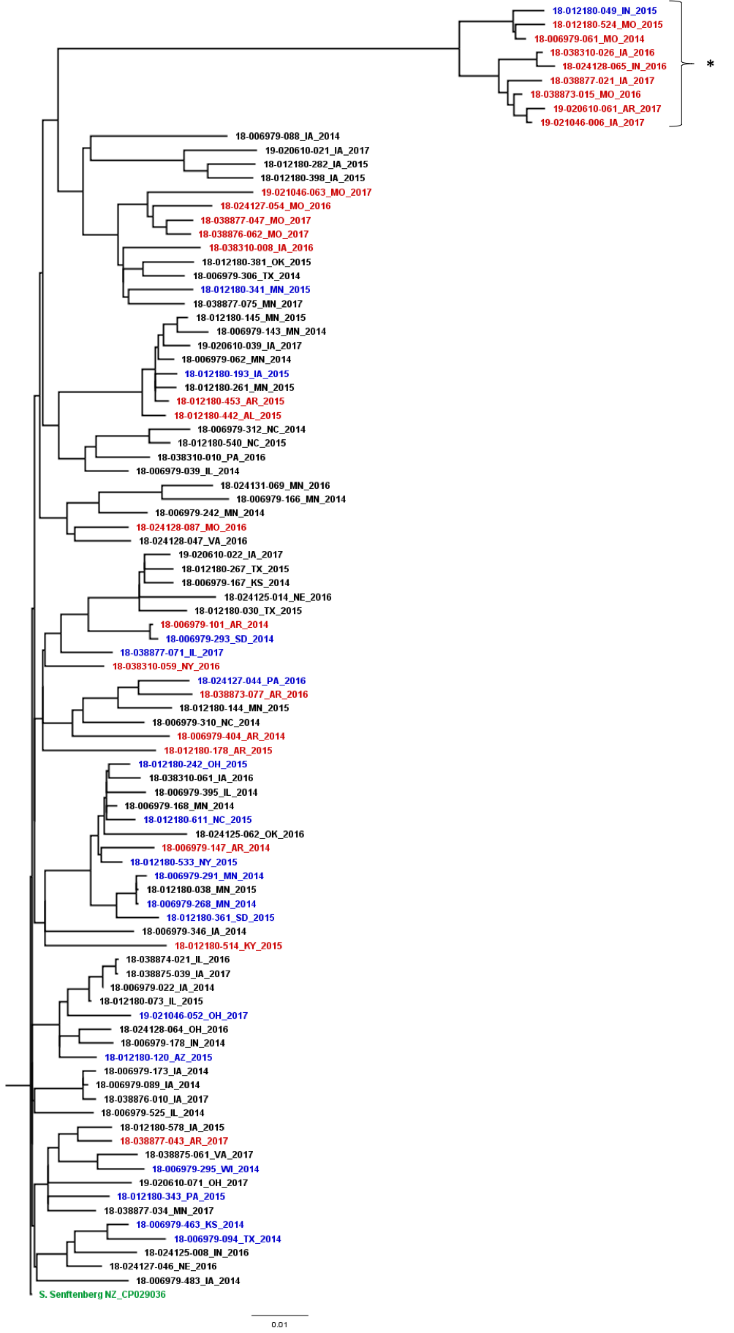
**

**Supplementary Figure 2.** The vSNP tree for ST14 clade shows a well-defined cluster with 9 isolates (8 from poultry and one from cattle) with the same antimicrobial and plasmid profile; these isolates have accumulated an average of 30.8 SNPs from the most recent common ancestor (*). Isolates from swine are in black, isolates from poultry in red, isolates from cattle are in blue and the *Salmonella* Senftenberg reference strain is in green.
